# The Effect of Mixtures and Additives on Dissolving Surfactant Lamellar Phases

**DOI:** 10.1021/acsphyschemau.4c00013

**Published:** 2024-07-15

**Authors:** Mitha Aljabri, Thomas Rodgers

**Affiliations:** † Department of Engineering, College of Engineering and Technology, University of Technology and Applied Sciences, Suhar OM 311, Oman; ‡ Department of Chemical Engineering, The University of Manchester, Manchester M13 9PL, U.K.

**Keywords:** surfactant mixtures, oil, DPD, diffusion, cluster size

## Abstract

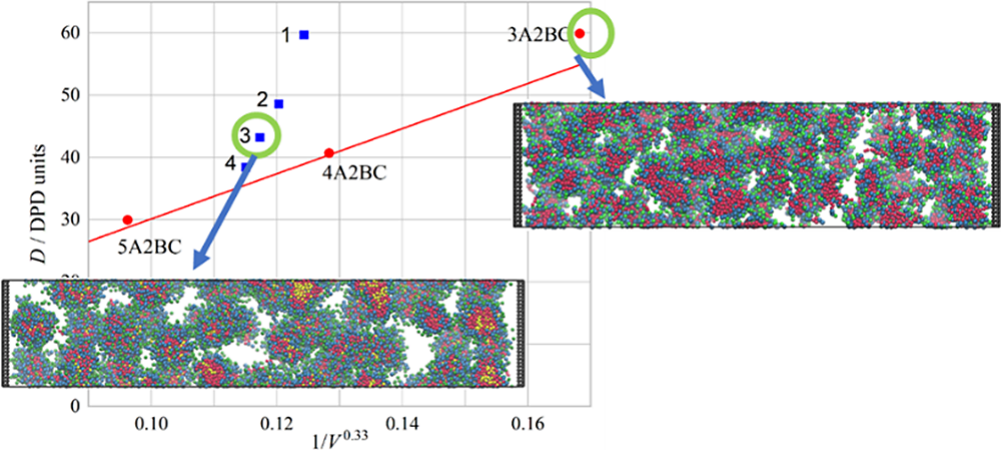

Understanding the dissolution process of surfactant solutions is important in formulating product design processes. The main goal of this study is to gain further insights into how additives and mixtures affect surfactant dissolution processes. To achieve this goal, dissipative particle dynamic simulations were used. Lamellar phases at 80% volume of surfactant were initially equilibrated with water. After reaching an equilibrium state, the dissolution simulations were carried out for different surfactant mixtures. To track the dissolution process, different metrics were used, including visual analysis, local concentration analysis, diffusion, and cluster size calculations. Results show that by having a mixture of surfactants, the diffusion of the micelles is not affected only by the size of the micelles as in pure surfactant systems, but there is also an effect due to the composition of the micelles. When oil is added to a surfactant system, the system acts like a longer chain surfactant system, but only when the chain of oil becomes sufficiently long.

## 1 Introduction

Surfactants are amphiphilic molecules that consist of at least two parts: a hydrophilic (water-liking) head part and a hydrophobic (water-hating) tail part.^1^ They are classified according to their headgroup’s charge: nonionic, zwitterionic, anionic, and cationic.^2^ Because of the unique chemical and physical properties of surfactant molecules, they have many applications in industries such as food, personal care, petroleum, and plastic.^1,3^ In aqueous solutions, when the surfactant concentrations exceed the critical micelle concentration (CMC), surfactants self-assemble into micelles varying in shape and size which coalesce as the surfactant concentration increases.^4−6^ At high surfactant concentrations, surfactant molecules in solution aggregate into lyotropic mesoscopic aggregates (e.g., hexagonal or lamellar phases).^7^ The process of dissolution of concentrated surfactant solutions in various concentrations of solvents has many applications in everyday usage of surfactant-containing products and in industrial processing and manufacturing of surfactant formulations; thus, understanding this process is very important.^8^ Surfactant dissolution has been studied experimentally and by simulation. Surfactant dissolution is studied experimentally by solvent penetration experiments, where the concentrated surfactant is placed in a capillary tube and allowed to come in contact with the solvent. A continuous concentration profile perpendicular to the interface appeared upon interdiffusion, and therefore the formed mesophases in this region can be analyzed using analytical techniques such as X-ray scattering, IR spectroscopy, and polarizing microscopy.^7^

To understand the early stages of the surfactant dissolution process, it is very difficult to access the time scale of seconds or less experimentally due to the small changes in surfactant mesophases appearing in this short time. To solve this problem, computer simulations are used to obtain valuable insights.^8^ There are different computer simulation techniques used to study surfactant aggregation and micelle formation and destruction, for example: molecular dynamics, Monte Carlo, and dissipative particle dynamics (DPD).^9^ DPD simulation is based on the simulation of soft beads in which their motion is governed by Newton’s laws of motion.^10^ Warren and coworkers simulate surfactant dissolution by placing two simulation boxes next to each other, one containing an equilibrated fluid of pure dimers and one with equilibrated solvent particles, and allowing them to interdiffuse. It was found that in a similar fashion to real systems, mesophases start to form at the interface. It was also found that the process is diffusion-controlled and adiabatic as soon as the mesophases start to form with similar time scales as in real systems, i.e., of order 10 μs.^11^

In this present study, we are looking to investigate the dissolution process of surfactant mixtures using DPD. Our objectives are to understand how adding different types of surfactants will affect the dissolution process. We also want to examine the dissolution process of mixtures of surfactant with oil. Several simulations and calculations were carried out for different surfactant mixes in order to achieve these aims; hence, the conclusions found here will be useful for other surfactant systems. The two surfactant systems under study consist of (1) 6 beads molecules—3A2BC and 4ABC—mixed in different proportions, and (2) a surfactant system with 6 bead molecules—3A2BC—of surfactant fixed and vary oil molecules ranging from one bead up to four beads. The DPD parametrization model will be developed based on Vishnyakov et al.^12^ To do so, this paper is structured as follows: In Section 1, an introduction about the surfactant dissolution process is given. Then, in Section 2, a brief introduction about the DPD method and the system parametrization is provided. In Section 3, the calculations used and results are presented and explained in detail. Finally, in Section 4, conclusions and findings are presented to be considered for future steps.

## 2 Methodology and Computational Details

### 2.1 DPD Algorithm

DPD is a type of coarse-grained model that was initially introduced by Hoogerbrugge and Koelman in 1992.^13^ In DPD simulations, the particles do not represent individual atoms or molecules, but each bead represents either a cluster of atoms or molecules or a fluid package.^14,15^ These beads interact with each other through a sum of three effective pairwise potentials: conservative, dissipative, and random represented as , , .

For any DPD particle *i*, the Newtonian equation of motion can be written as,^14^
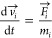
1
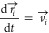
2
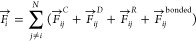
3

These forces between the *i* particle and the *j* particle are zero beyond a certain cutoff radius which is always used as a reduced unit of length, *r*_c_ = 1.

The conservative force is a soft repulsive force which determines the thermodynamics of the DPD system and is given by^16^
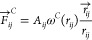
4where *A*_*ij*_ is the maximum repulsion strength between a pair of particles *i* and *j*, and .

The dissipative force () describes the friction and energy dissipation as one bead moves past another. It is given by^17^
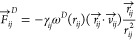
5where γ_*ij*_ is the friction coefficient, ω^*D*^ is the dissipative weight function, and  is the relative velocity of the two beads.

The random force () is introduced to counterbalance the dissipation and inject energy back into the system^17^
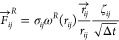
6where σ_*ij*_ is the noise strength, ω^*R*^ is the random weight function, ζ_*ij*_ is a random variable with a Gaussian distribution,^1^ and Δ*t* is the time step.^18^ To satisfy the fluctuation–dissipation theorem, ω^*C*^, ω^*D*^, ω^*R*^, γ_*ij*_, and σ_*ij*_ must follow these relationships,
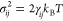
7
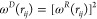
8where the switching functions are often defined as
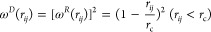
9

The dissipative and random forces act as a thermostat. Surfactant beads are connected by harmonic springs between beads where the harmonic force is given by^13^
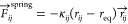
10where κ_*ij*_ is the spring force constant and *r*_eq_ is the equilibrium bond length.^19^

### 2.2 Parametrization

In this current study, we have simulated several mixtures of surfactant systems to understand how adding different materials affects the lamellar phase dissolution process, compared to pure systems. In this model, two different materials were mixed in different proportions and then allowed to dissolve together. Surfactant molecules are modeled as connected beads of the hydrophobic tail (A beads) and the hydrophilic head (B and C beads). Water molecules are treated as individual DPD beads (W beads), and oil molecules are treated as separate DPD bead chains of different lengths (O beads).

The base molecule in this study was represented by a chain of beads (AAABBC) consisting of a block of three A beads (−C_3_H_6_−) representing the hydrophobic part of the surfactant, connected to two B beads (−EO_2_−) as part of the hydrophilic head and one C bead (−OSO_3_^–^) as the other part of the hydrophilic head. In this case, as only a general effect of additives and surfactants is explored, an explicit charge is not added; however, the effect of an increased hydrophilic interaction is captured with a lower repulsion parameter for the C-type bead. This approach has been shown to be affective in previous work.^20,21^

The W-type beads each represent four water molecules thus the mass of each bead is equal to 1.2 × 10^–25^ kg. To reproduce the dimensionless compressibility of water, the self-repulsion parameter must therefore be taken as 106.5.^22^ To match the dimensionless density of the simulations to the real density of water, the value of the bead radius, *r*_c_, is equal to 7.1 × 10^–10^ m. The O-type beads represent oil (−C_3_H_6_−). The bead–bead repulsion parameters were taken from Vishnyakov et al.,^12^ where these parameters reproduce accurate CMCs for the surfactants studied; when we applied them in this study, lamellar phases were produced at the expected concentrations. As the simulation is taken at standard room temperature then *T* = 298 K. Table 1 shows the interaction parameters between beads used in this study, and a summary of the simulation parameters is given in Table 2.

**Table 1:** Interaction Parameters Used in This Study

	A	B	C	W	O
**A**	106.5	113.0	127.7	126.5	106.5
**B**	113.0	106.5	106.5	107.5	113.0
**C**	127.7	106.5	106.5	83.0	127.7
**W**	126.5	107.5	83.0	106.5	126.5
**O**	126.5	113.0	127.7	126.5	106.5

**Table 2:** Parameters Selected for DPD Model

parameter	value
time step, Δ*t*	0.02
cut-off distance, *r*_c_	1σ
reduced energy, *k*_B_*T*	1
density, ρ	3
friction coefficient, γ	4.5
bead radius, σ	1
spring force constant, κ_*ij*_	75
equilibrium bond length, *r*_eq_	0.45

### 2.3 Implementation

Simulations of micellar structures can be affected by the box size; hence, in this current study, large simulation boxes were used in order to accommodate a fixed number of surfactant molecules assembling into lamellar phases. The dimensions used for equilibrium simulations were 20 × 20 × 20*r*_c_^3^ with a simulated bead density of 3 beads/*r*_c_^3^.

The simulations were undertaken in two parts: the first part is the simulations to equilibrium, and then the second part is the dissolution simulation. For equilibrium simulations, a mixture of surfactant molecules and solvent were initially placed together in random configurations to form a concentration of 80% by volume of surfactant. The simulations were carried out in an NVT ensemble (constant number, volume, and temperature) using the DL_MESO software package version 2.6.^23^

When the equilibrium state was reached, a lamellar phase was formed inside the initial simulation box. Afterward, dissolution simulations were carried out with the original equilibrium simulation box stitched to a larger along the (*x*) axis to connect with the pure solvent. To do this, the equilibrium simulation boxes were rotated to orient vertically and cropped to only include four lamellar layers in the box. This box of rotated cropped lamellar layers is then stitched to an equilibrated water box and bound on both sides orthogonally to the *x*-axis with two rows of fixed beads as a wall with similar interaction parameters to the water beads. This causes the dissolution of the lamellar layers to occur only in one direction (*x*) and avoids a periodic boundary condition in this direction.

The wall repulsion potential is given by^8^
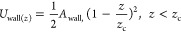
11where *z* is the distance between the particle and the boundary, *A*_wall_ is the DPD repulsive force magnitude, and *z*_c_ is the surface repulsion range.

**Figure 1:**
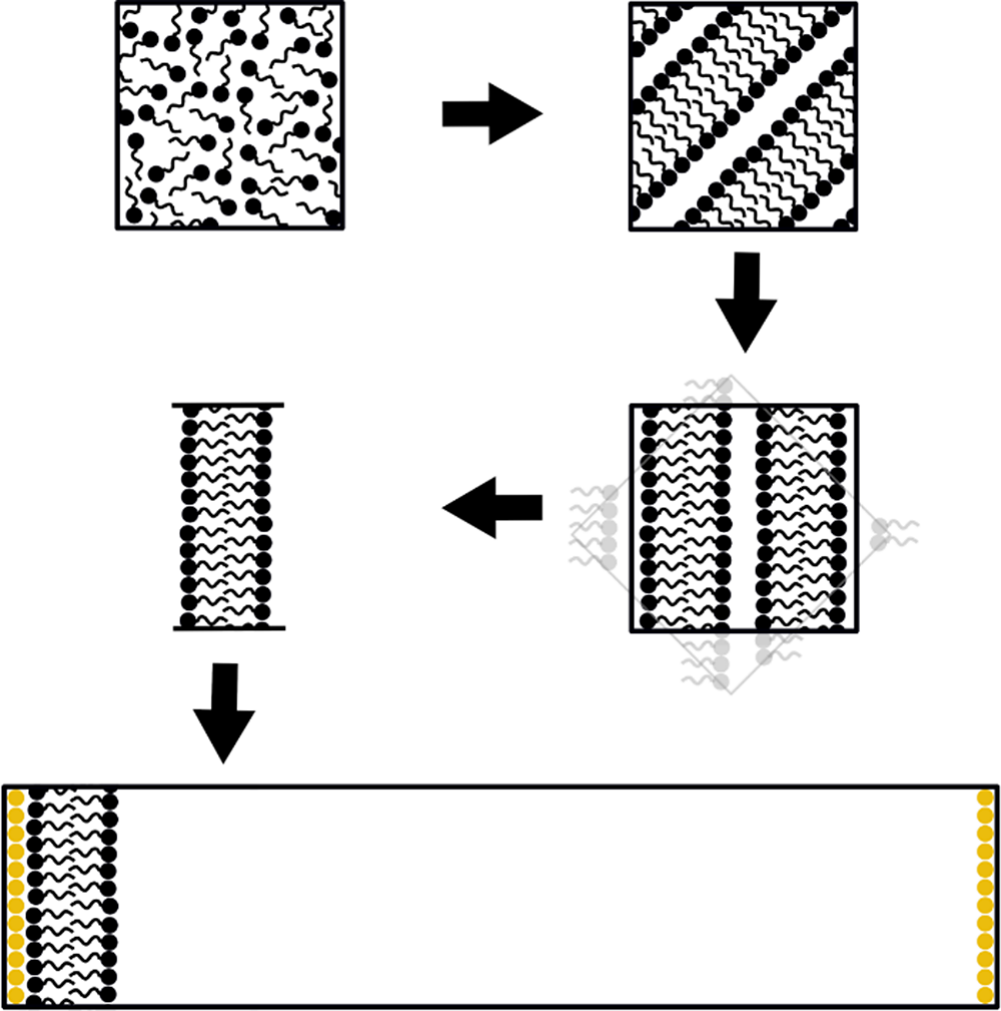
Schematic approach describing the simulation setup, water not shown. A randomized arrangement is equilibrated to a lamellar phase. This is rotated and cropped to align to a water box which is then added to the lamellar phase, with wall beads shown in yellow.

After the two boxes are attached together, the whole box is relaxed by restraining the *C* beads by a spring to their original location and running the DPD simulation for 10,000 steps. This allows any local variations in pressure and density happened by the stitching process to even out. Figure 1 represents the simulation setup used in this study.

In this paper, we examine two systems; the first is a mixture of surfactants 3A2BC and 4ABC, which are mixed in different proportions (0, 25, 50, 60, 70, 75, and 100%), while the second is the surfactant system AAABBC mixed with oil chains represented by different number of beads, varying from one bead up to four beads (70% surfactant, 10% oil). For all mixture systems studied, the end concentration was fixed at 20%. This enables the comparison between different surfactant system mixtures. To achieve this, the dissolving simulation boxes varied in size from 79 × 20 × 20*r*_c_^3^ (3A2BC) to 91 × 20 × 20*r*_c_^3^ (4ABC) for surfactant mixtures and to 99 × 20 × 20*r*_c_^3^ (3A2BC with 4 beads oil) for the surfactant/oil mixtures.

### 2.4 Analysis

A number of different methods were applied to analyze the results from the simulations of the dissolution process for various surfactant systems. The first was a visual analysis of the end systems visualized using Visual Molecular Dynamics (VMD) software.^24^ The second is via local concentration measurements along the *x*-direction (directions *y* and *z* were averaged over an *x* range of 0.332*r*_c_).

In previous work, a zonal model was developed.^21^ In this zonal model, the zone system values were equally divided into different numbers of zones, and by applying Fick’s Law, the diffusion parameters were obtained,
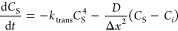
12
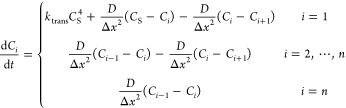
13where *k*_trans_ is the phase transformation constant, *D* the diffusion constant, *C*_S_ is the concentration in the surfactant lamellar phase zone, and *C*_*i*_ represents the concentration of the numbered zones. In this paper, a total of five zones were used.

For each micelle formed upon dissolution, the number of chains was calculated based on the MyGang algorithm.^25^ Cluster of three or fewer chains are not included in these calculations since they are free surfactants rather than micelles. This method was validated in reference (20).

## 3 Results and Discussion

### 3.1 Surfactant Mixtures

**Figure 2:**
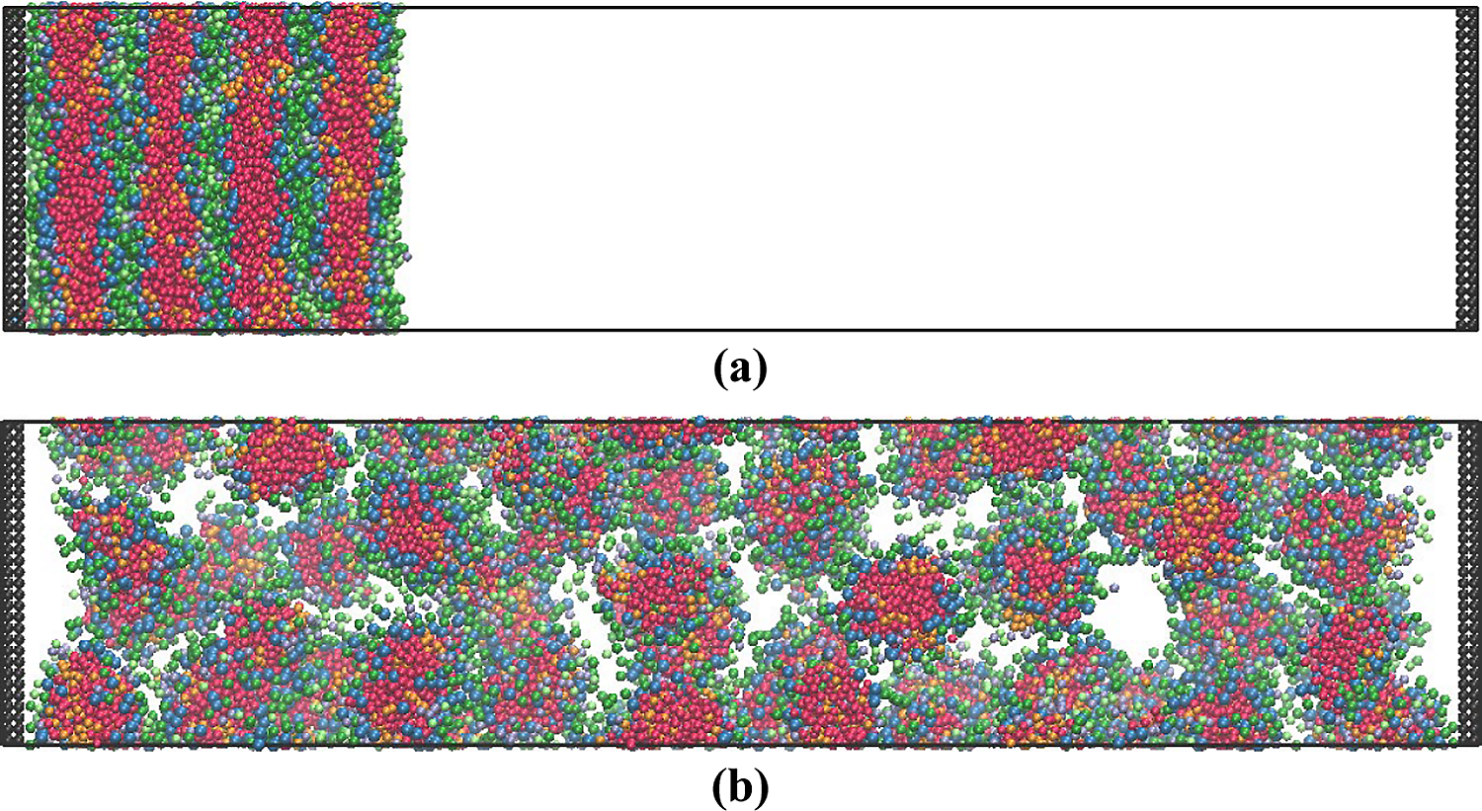
(a) Start point and (b) end point of the dissolution for the mixture of 75% 3A2BC/25% 4ABC. 3A2BC shown as A: red, B: blue, and C: green; and 4ABC shown as A: orange, B: light blue, and C: light green.

In all cases, the dissolution process starts by rearrangement of the lamellar layer closest to the water, micelles form by breaking off from the layer. This then allows the penetration of water into the first lamellar layer, which causes the lamellar layer to break into multiple spherical micelles. This process is repeated layer by layer through the lamellar structure. After the spherical micelles form, they diffuse along the length of the box until they are even distributed, as shown in Figure 2b. In this case, all the different mixtures produce spherical micelles. The two types of surfactant are evenly mixed with each other at all stages of the dissolution process (Figure 2).

**Figure 3:**
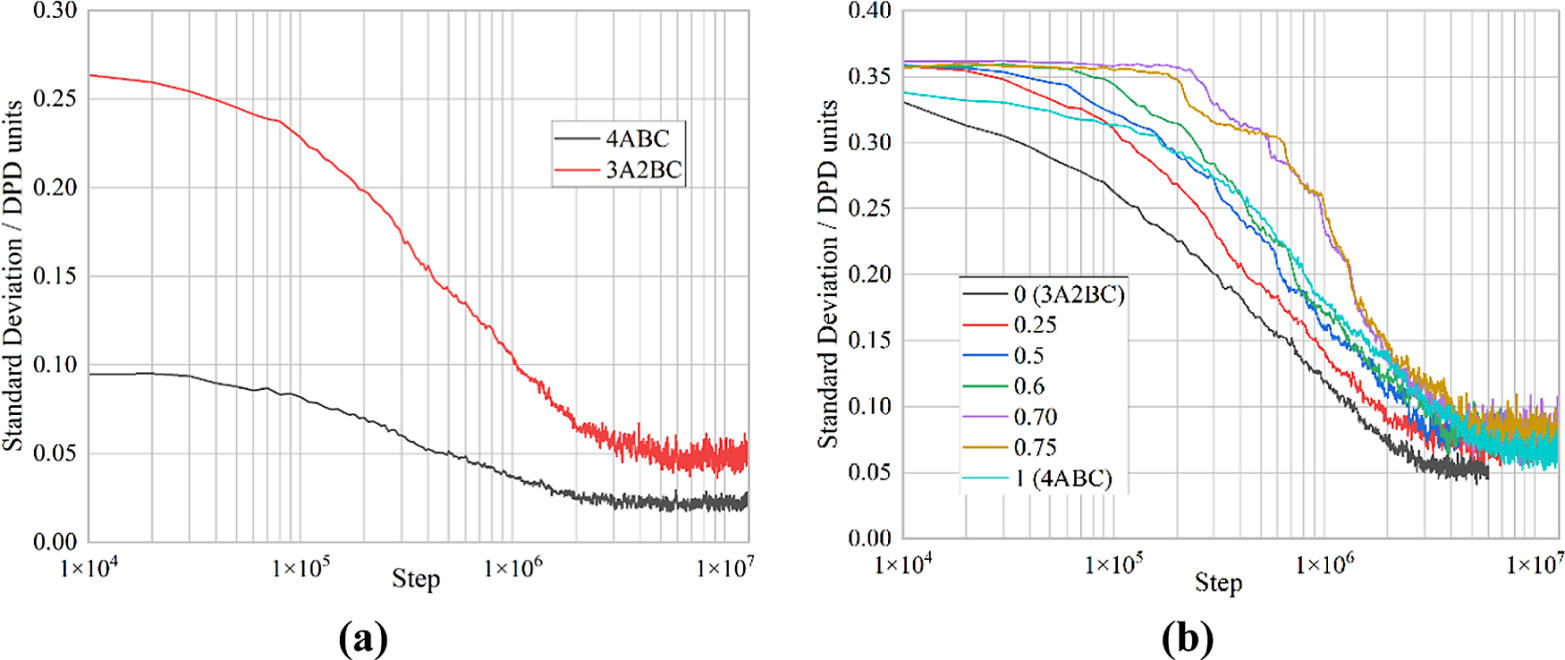
Standard deviation of concentration of surfactant in the simulation box (a) for each individual surfactant for the mixture of 75% 3A2BC/25% 4ABC and (b) for the total surfactant at the different mixture concentrations.

The dissolution process can be further investigated by analyzing the concentration of the surfactant at different points of the box until it is dissolved completely and the box becomes homogeneous. Figure 3 shows the standard deviation of the surfactant concentration across the box. The higher the value, the more nonhomogenous the system is, i.e., the surfactant is distributed at one end of the box. Figure 3a shows the results for a mixture of 75% 3A2BC with 25% 4ABC. Since there is less 4ABC in the system, the standard deviation is lower; however, the same profile shape is obtained for both the 3A2BC and the 4ABC surfactants when calculated individually. The two surfactant types are evenly mixed in both the lamellar phases and the final micelles, and thus the total surfactant concentration can be analyzed rather than the two surfactants separately.

Figure 3b shows the standard deviation of the total surfactant concentration across the box. As the initial concentration of the total surfactant is the same in all cases, the initial value of the standard deviation is the same, and as the end value is also the same, the final value of the standard deviation is also approximately the same. There are some minor variations due to slightly different box sizes.

As the proportion of the 4ABC surfactant increases, the dissolution process slows down, and the time until the dissolution process starts, i.e., the breakup of the first lamellar sheet, increases, apart from for the pure 4ABC system, where this initial breakup occurs more quickly. The gradient of the falling part of the curve then relates to the continued transformation of the lamellar phase to micelles and the micelles diffusing through the box. This form of result is difficult to analyze due to the long-time scales and the interplay between the two aspects.

**Figure 4:**
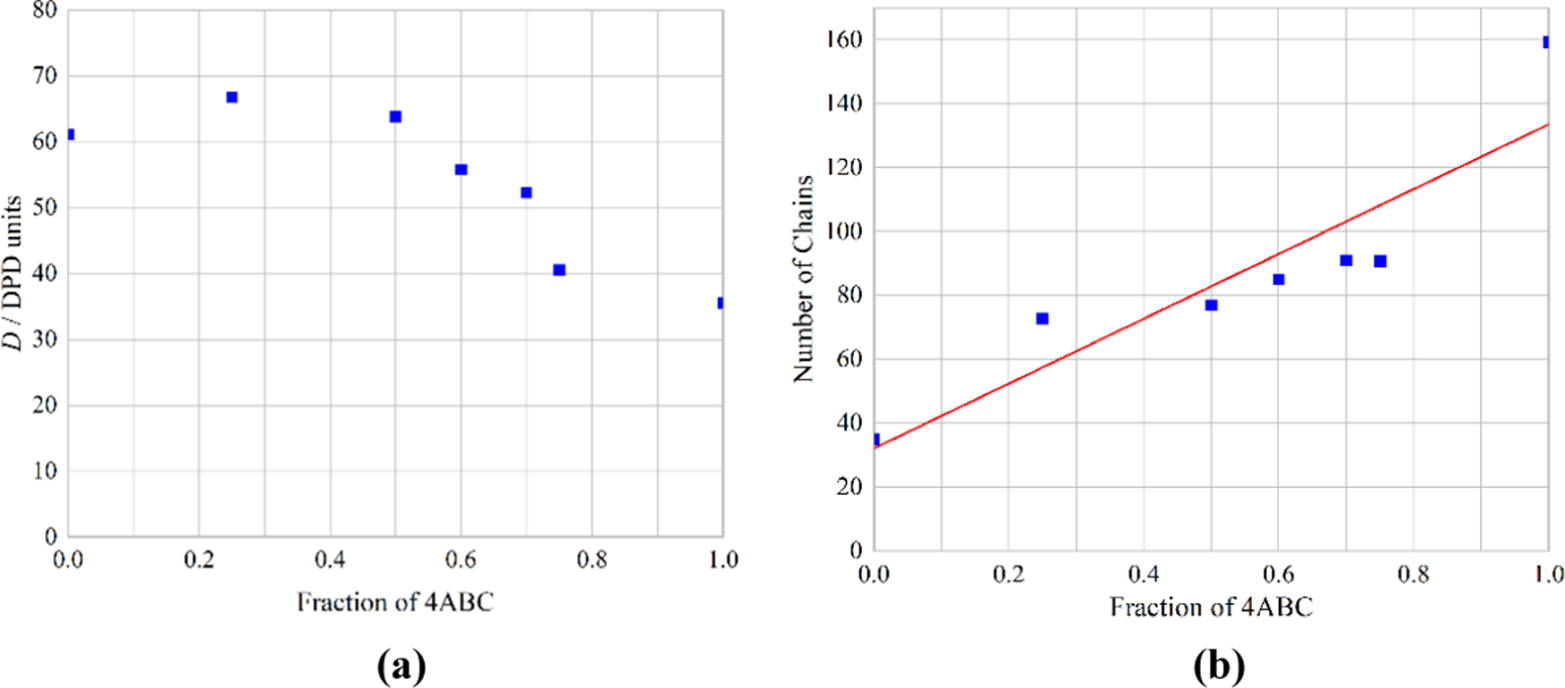
Variation of (a) the diffusion coefficient and (b) the average number of chains in each micelle with change in the surfactant fraction. The red line is a linear best fit for comparison.

Therefore, the best option for analysis is to split the dissolution into two stages as undertaken with the zonal model, eqs 12 and 13. In this case, we will focus on the diffusion constant, *D*, and analyze the variation with the change in surfactant fraction. From Figure 4a, it is clear that there is a nonlinear variation of the diffusion with the surfactant fraction. Concentrations of up to 50% of 4ABC seem to have very little effect on the diffusion constant, and then again, concentrations above 75% of 4ABC also have little effect, albeit the constant is at a lower value. This means that the majority of the change occurs between 50 and 75%.

The other key parameter for the micelles is their size. In Figure 4b, this is represented by the average number of chains in each micelle. As the fraction of the 4ABC surfactant increases, so does the average number of chains in each micelle. This is likely due to the fact that the 4ABC has a relatively small headgroup (BC compared with the chain of A) so favors less curvature, otherwise which is produced with large micelles. This change in the number of surfactant chains is approximately linear with the surfactant fraction.

Table 3 summarizes the results from Figure 4 and provides the corresponding value of 1/*V*^0.33^ for each system. This is the reciprocal of the average volume of the micelles to the power of a third, which, according to Stokes–Einstein diffusion for the solid sphere, is related to the diffusion constant. In previous work,^20^ this relationship works for pure surfactant systems of this type.

**Table 3:** Summary of the Cluster Size Calculations for the Mixed Surfactant Systems

fraction	diffusion	no. of chains	1/*V*^0.33^	number of micelles
0.00 (3A2BC)	61.1	35.0	0.168	91
0.25	66.8	72.7	0.132	44
0.50	63.8	76.9	0.129	42
0.60	55.8	85.0	0.125	38
0.70	52.2	91.0	0.122	35
0.75	40.5	90.8	0.122	35
1.00 (4ABC)	35.5	159.3	0.102	20

**Figure 5:**
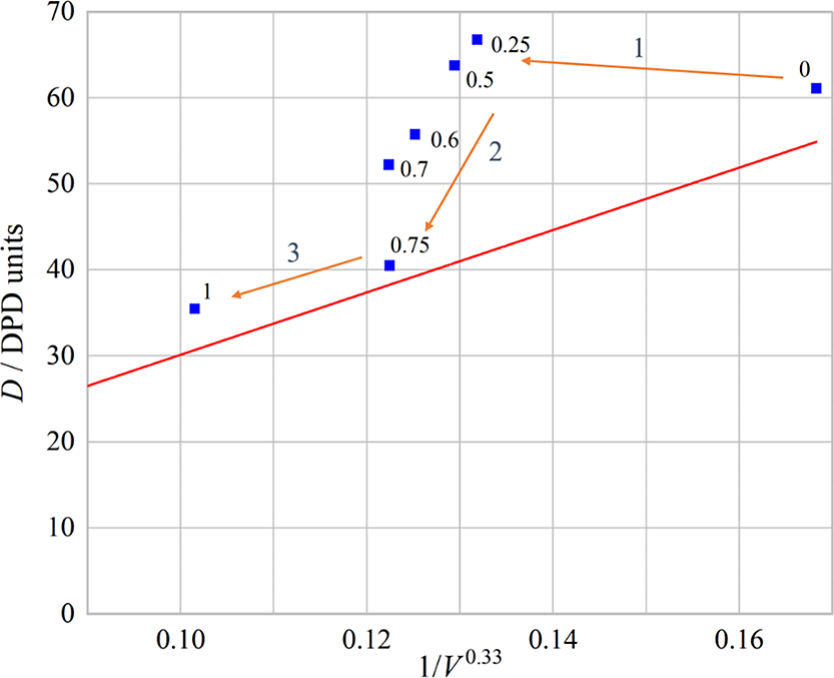
Diffusion *D* vs 1/*V*^0.33^ for all the mixed surfactant systems. The red line is the trend line from previous work^20^ for pure surfactant systems. The fractions refer to the fraction of 4ABC in the mixture.

Figure 5 shows the variation of the diffusion constant with 1/*V*^0.33^ with the pure surfactant line shown as the red line.^20^ As the proportion of the 4ABC surfactant increases, an interesting pattern forms in place of the pure surfactant line. This is likely because with surfactant micelles, there are two effects controlling the diffusion constant. The first is merely the size of the micelles, with larger micelles diffusing more slowly, and the second is the interaction between micelles, something that the Stokes–Einstein equation does not take into account. This dependency on the interaction has been seen experimentally with both increased and decreased diffusion.^26^ In the pure surfactant systems, the micelle size and the interactions are directly controlled just by the single surfactant; however, for mixed systems, the micelle sizes and interactions can be controlled separately.

As a small amount of 4ABC is mixed into the system, the formed micelles increase in size; in fact, the number of surfactant chains per micelle more than doubles by the time 50 vol % of the composition is 4ABC. This increase in size is, however, not accompanied by the expected reduction in the diffusion constant, region 1 as shown in Figure 5. This is likely due to the fact that the curvature of the micelles is greatly influenced by the addition of the 4ABC surfactants, but the interactions between the micelles are still dominated by the 3A2BC surfactants.

As the concentration of 4ABC continues to increase from 50 to 75 vol %, the micelle size continues to grow as expected, but the diffusion constant also reduces, region 2 as shown in Figure 5. This is likely due to the fact that the higher concentration of 4ABC surfactant has an effect on the interactions between the micelles, likely reducing the repulsions between them.

When the micelles are mostly 4ABC (over 75 vol %), they behave more like pure 4ABC micelles in terms of diffusion constant, but as the fraction of 3A2BC increases, the micelle size reduces due to the 3A2BC trying to increase the micelle curvature with its relatively large head size, region 3 on Figure 5.

### 3.2 Surfactant Systems with Oil

**Figure 6:**
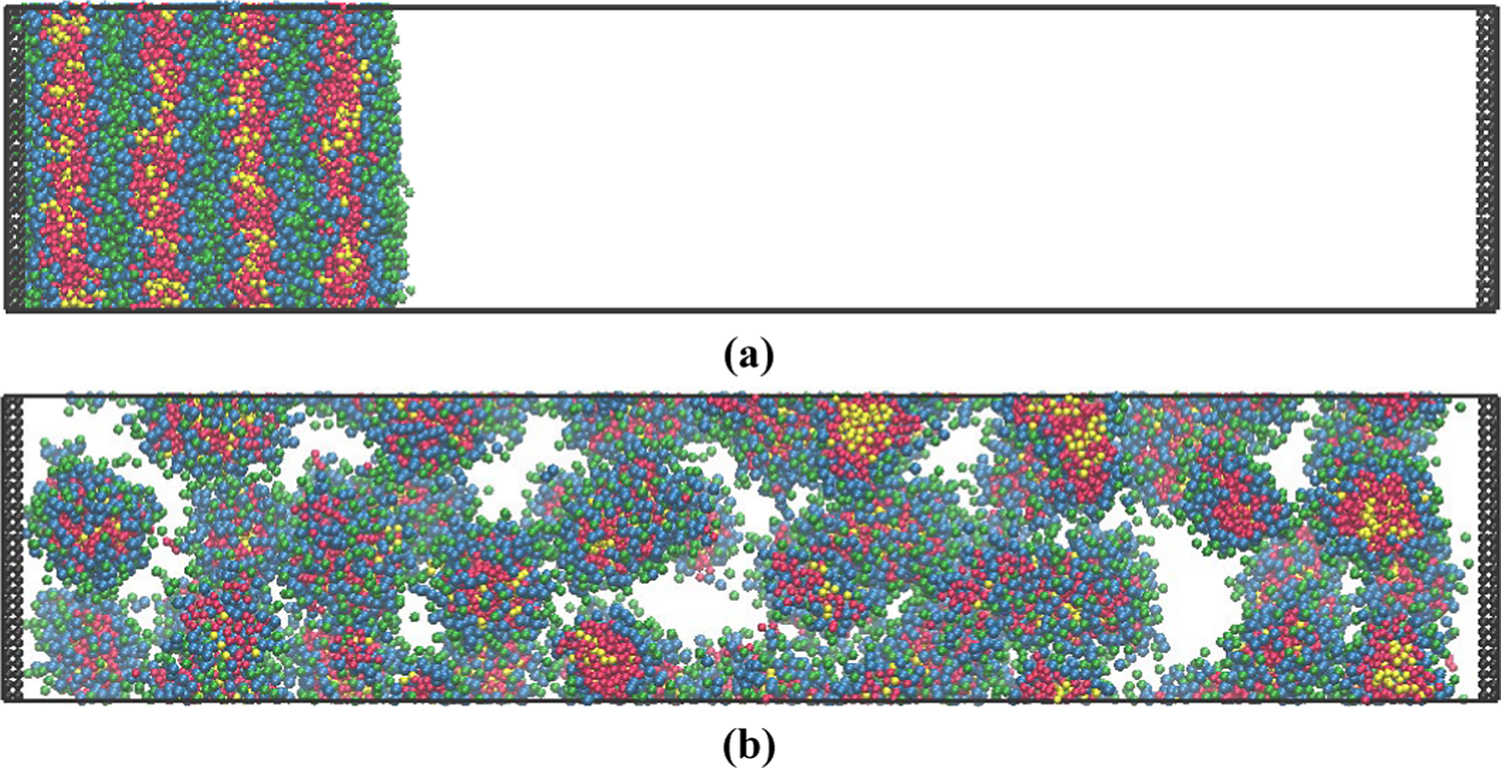
(a) Start point and (b) end point of the dissolution for the 3 bead oil system with a surfactant. 3A2BC shown as A: red, B: blue, and C: green; and the oil shown as O: yellow.

Similarly, in the cases with mixed surfactant systems, the dissolution process with added oil started by rearrangement of the lamellar layer closest to water with the formation of micelles which then break off from the layer. This then allows the penetration of water into the first lamellar layer which breaks into multiple spherical micelles. The oil molecules randomly enter the micelles during the breaking process. After the spherical micelles form, they diffuse along the length of the box until they are evenly distributed, as in the example shown in Figure 6. In this case, all of the different oil chain lengths produce spherical micelles.

**Figure 7:**
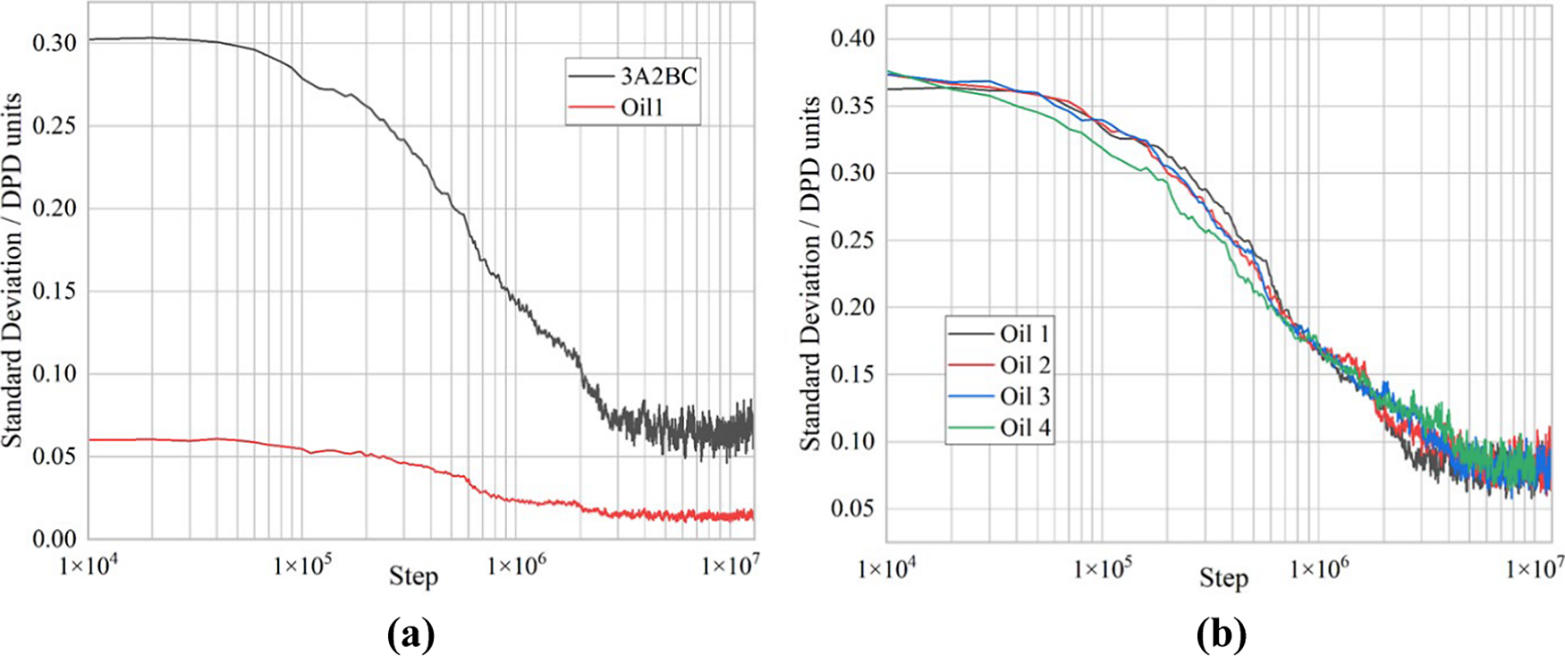
Standard deviation of concentration of surfactant in the simulation box (a) for the surfactant and the oil for the mixture the 1 bead oil and (b) for the total surfactant and oil with the different oil bead lengths.

The dissolution process can be investigated further by analyzing the concentration of the surfactant and oil at different points of the box until the surfactant is dissolved completely in the box and the box becomes homogeneous. Figure 7 shows the standard deviation of the surfactant concentration across the box. The higher the value, the more nonhomogenous the system is, i.e., surfactant is distributed at one end of the box. Figure 7a shows the results for a mixture of 3A2BC consisting of single bead. As less oil is included in the system, the standard deviation reduces; however, the same profile shape is obtained for both the 3A2BC and oil when calculated individually. The surfactant and oil are evenly mixed in both the lamellar phases and the final micelles, and thus the total concentration can be analyzed instead of the two separate concentrations.

Figure 7b shows the standard deviation of the total concentration across the box. As the initial concentration of the surfactant and oil is the same in all cases, the initial value of the standard deviation is the same, and as the end value is also the same, the final value of the standard deviation is also approximately the same. There are some minor variations due to different box sizes.

As the length of the oil molecule increases, the dissolution process slows down, and while the time until the dissolution process starts, i.e., the breakup of the first lamellar sheet, remains similar, the gradient of the falling part of the curve related to the continued transformation of the lamellar phase to micelles and the micelles diffusing through the box becomes shallower. This means that the diffusion of the micelles likely slows down; however, this form of result is difficult to analyze due to the long-time scales and the interplay between the two aspects.

**Figure 8:**
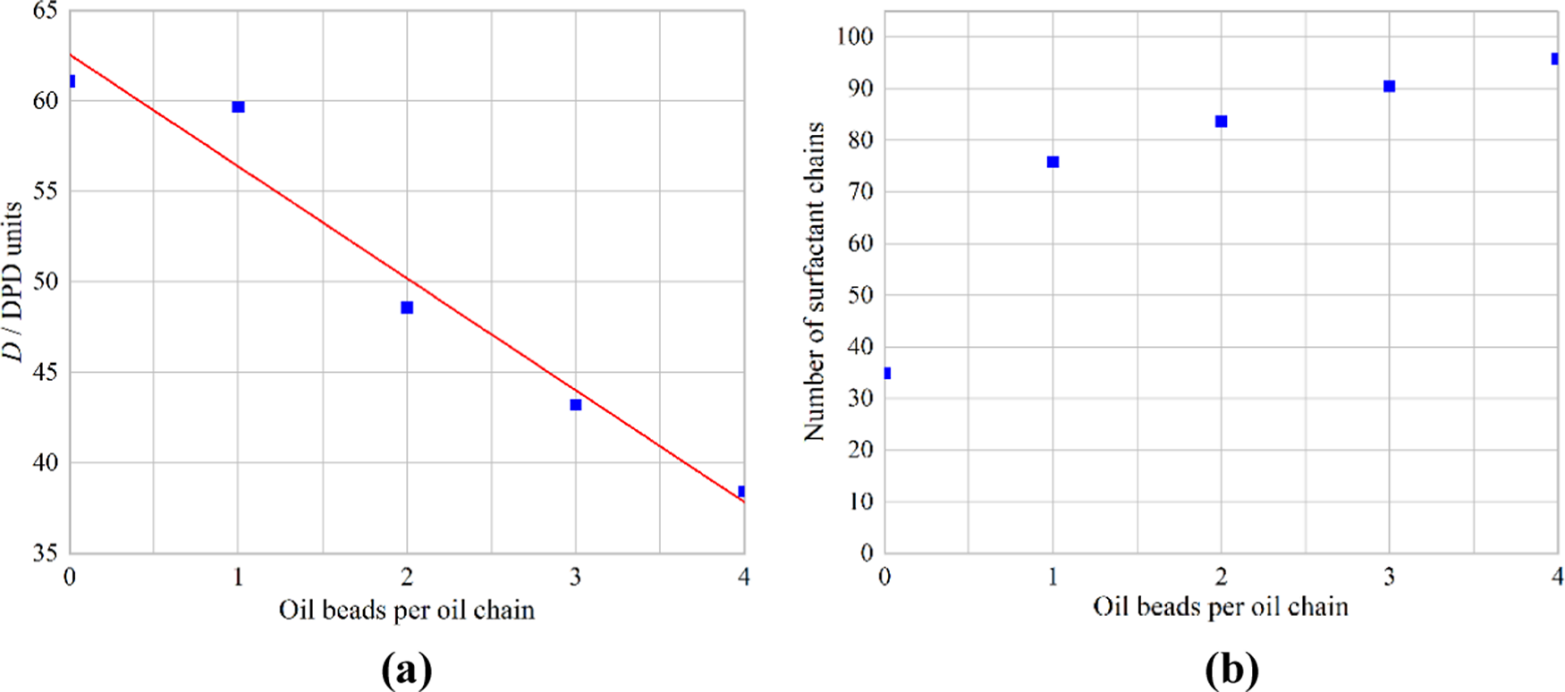
Variation of (a) the diffusion coefficient and (b) the average number of chains in each micelle with change in the surfactant fraction. The red line is a linear best fit for comparison.

Therefore, the best option to analyze this is to split the dissolution into two stages as undertaken by the zonal model, eqs 12 and 13, for the surfactant mixtures. We will again focus on the diffusion constant, *D*, and analyze the variation with the change in the surfactant fraction. From Figure 8a, it is clear that there is an approximately linear reduction of the diffusion with the length of the oil molecule.

The other key parameter of the micelles is their size. In Figure 8b, this is represented by the average number of surfactant chains in each micelle. As the oil molecule length increases, so does the average number of surfactant chains in each micelle, although this effect seems to diminish with increasing oil chain length. This is likely due to the fact that the oil molecules only fill the centers of the micelles, and thus more surfactant is needed to “wrap around” the oil chains.

**Figure 9:**
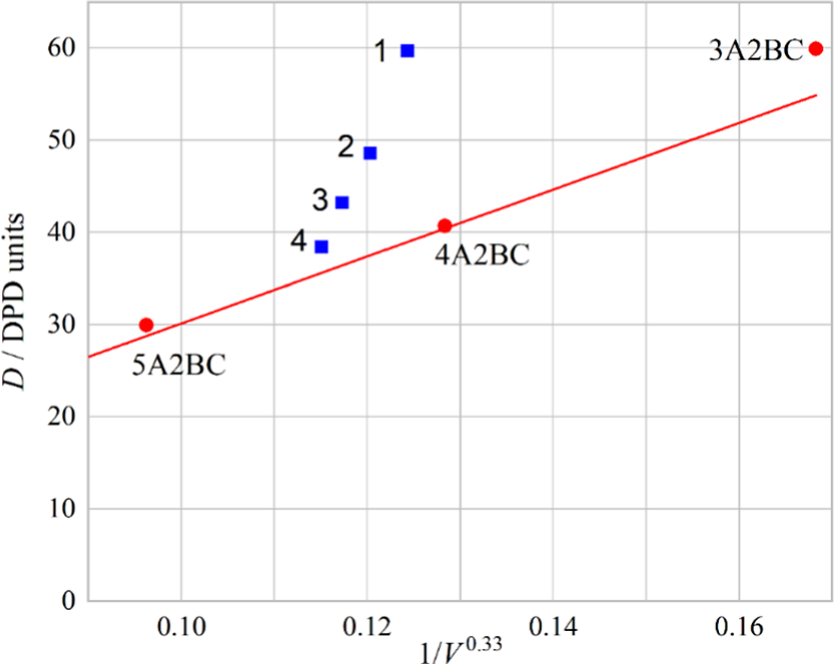
Diffusion *D* vs 1/*V*^0.33^ for all surfactant-oil mixtures. The red line is the trend line from ref (20) for pure surfactant systems. The numbers refer to the number of oil beads in the chain.

Table 4 summarizes the results from Figure 8 and provides the corresponding value of 1/*V*^0.33^ which includes the oil chains for each system. Figure 9 shows the variation of the diffusion constant with 1/*V*^0.33^ with the pure surfactant line shown as the red line from.^20^ As the length of the oil molecule increases, an interesting pattern appears that deviates from the pure surfactant line. Again, this is likely due to the two effects controlling the diffusion constant. The first is merely the size of the micelles, with larger micelles diffusing slowly, and the second is the interaction between the micelles, which the Stokes–Einstein equation does not consider.

**Table 4:** Summary of the Cluster Size Calculations for the Mixed Surfactant Systems^a^

system	diffusion	no. of chains	1/*V*^0.33^	number of micelles
3A2BC only	61.1	35.0	0.168	91
1 bead oil	59.7	75.9	0.124	42
2 bead oil	48.6	83.7	0.120	38
3 bead oil	43.2	90.4	0.117	35
4 bead oil	38.4	95.7	0.115	33

a The number of chains only includes the surfactant chains, while the micelle volume includes the oil chains within the micelles.

As the oil molecules are added to the system, the formed micelles increase in size with the number of surfactant chains per micelle more than doubling. This increase in size is, however, not accompanied by the expected reduction in the diffusion constant (Figure 9). This is likely due to more surfactant chains being needed to surround the oil molecules, making the micelles increase in size, but the interactions between the micelles are still only caused by the 3A2BC surfactant.

As the length of the oil molecules increases, the micelles start to act less like a filled micelle and more like a micelle created by a surfactant with a longer hydrophobic tail, i.e., it approaches to the red line in Figure 9. This is likely due to the fact that as the oil chains increase in length, they become less mobile within the micelle and form stronger interactions with the hydrophobic tails of the surfactants.

## 4 Conclusions

The dissolution process for different surfactant mixtures was studied using DPD simulations. Several metrics were used to give further insights into how surfactant mixtures are dissolved, including visual analysis, concentration analysis, cluster size, and diffusion calculations. Visual analysis showed that all of the different surfactant mixtures studied break into spherical micelles. From the concentration analysis, it was found that by having a mixture of surfactants, the different compositions affect the two parts of the process, the transformation, and the micelle diffusion—identified in previous work^21^—in a complex manner.

Mixing surfactant systems causes the sizes and the diffusion constant of the micelles to change independently. This is because the mixture effects the curvature of the micelles and the interactions of the micelles differently. Small additional amounts of a longer chain surfactant added have more effect on the curvature of the micelle rather than the interactions between the micelles. This means that by mixing surfactants, it is possible to create larger micelles that diffuse more quickly than expected, or vice versa.

By adding long chains of oil, the surfactant system starts to act more like a longer-chained surfactant system. If the oil chains are shorter, they make the micelles larger to physically fit the oil inside the micelles, but they have no effect compared with the oil-free micelles in terms of do not appreciatively change the dissolution process. This means that small hydrophobic molecules can be added to micelles without affecting their diffusivity.
